# Otoacoustic Emissions for Outcome Prediction in Postanoxic Brain Injury

**DOI:** 10.3389/fneur.2018.00796

**Published:** 2018-09-25

**Authors:** Daniel Kondziella, Anne Marie Jensen, Thomas Hjuler, Michael Bille, Jesper Kjaergaard

**Affiliations:** ^1^Department of Neurology, Rigshospitalet, Copenhagen University Hospital, Copenhagen, Denmark; ^2^Department of Otorhinolaryngology, Head and Neck Surgery and Audiology, Rigshospitalet, Copenhagen University Hospital, Copenhagen, Denmark; ^3^Department of Cardiology, Rigshospitalet, Copenhagen University Hospital, Copenhagen, Denmark

**Keywords:** anoxic-ischemic encephalopathy, brain edema, cardiac arrest, prognostication, outcome

## Abstract

**Background:** Non-invasive, easy-to-use bedside tools to estimate prognosis in unresponsive patients with postanoxic brain injury are needed. We assessed the usefulness of otoacoustic emissions as outcome markers after cardiac arrest.

**Methods:** Distortion product otoacoustic emissions (DPOAE) and transient evoked otoacoustic emissions (TEOAE) were measured in cardiac arrest patients whose prognosis was deemed to be poor following standard neurological assessment (*n* = 10). Ten patients with myocardial infarction without prior loss of consciousness served as controls.

**Results:** Compared to controls with myocardial infarction, cardiac arrest patients with poor neurological prognosis had significantly less often preserved DPOAE (9.2 vs. 40.8% positive measurements; OR 0.15 (CI 0.07–0.30); *p* < 0.0001). Partially preserved DPOAE were noted in 4 cardiac arrest patients. TEOAE were not statistically different between the two groups.

**Conclusions:** Despite their convenience, otoacoustic emissions cannot be used as reliable prognostic markers in cardiac arrest survivors. This is because we identified 4 cases with partially preserved otoacoustic emissions in a sample of 10 unresponsive post-cardiac arrest patients whose neurological condition was so poor that active treatment was withdrawn. However, we suggest that future research should address if decaying outer hair cell function over time may serve as a proxy for evolving ischemic brain damage.

## Introduction

Otoacoustic emissions are small sounds generated by the outer hair cell activity in the cochlear and can be measured in the ear canal of healthy people. These sounds are by-products of active processes in the cochlea, in which motility of the outer hair cells adjusts the basilar membrane and amplifies weak sounds. Although they do not contribute to hearing, otoacoustic emissions are clinically important because they allow evaluation of the integrity of outer hair cell function and the cochlea. They are therefore routinely assessed for evaluation of hearing, including screening in newborns.

**Figure 1:**
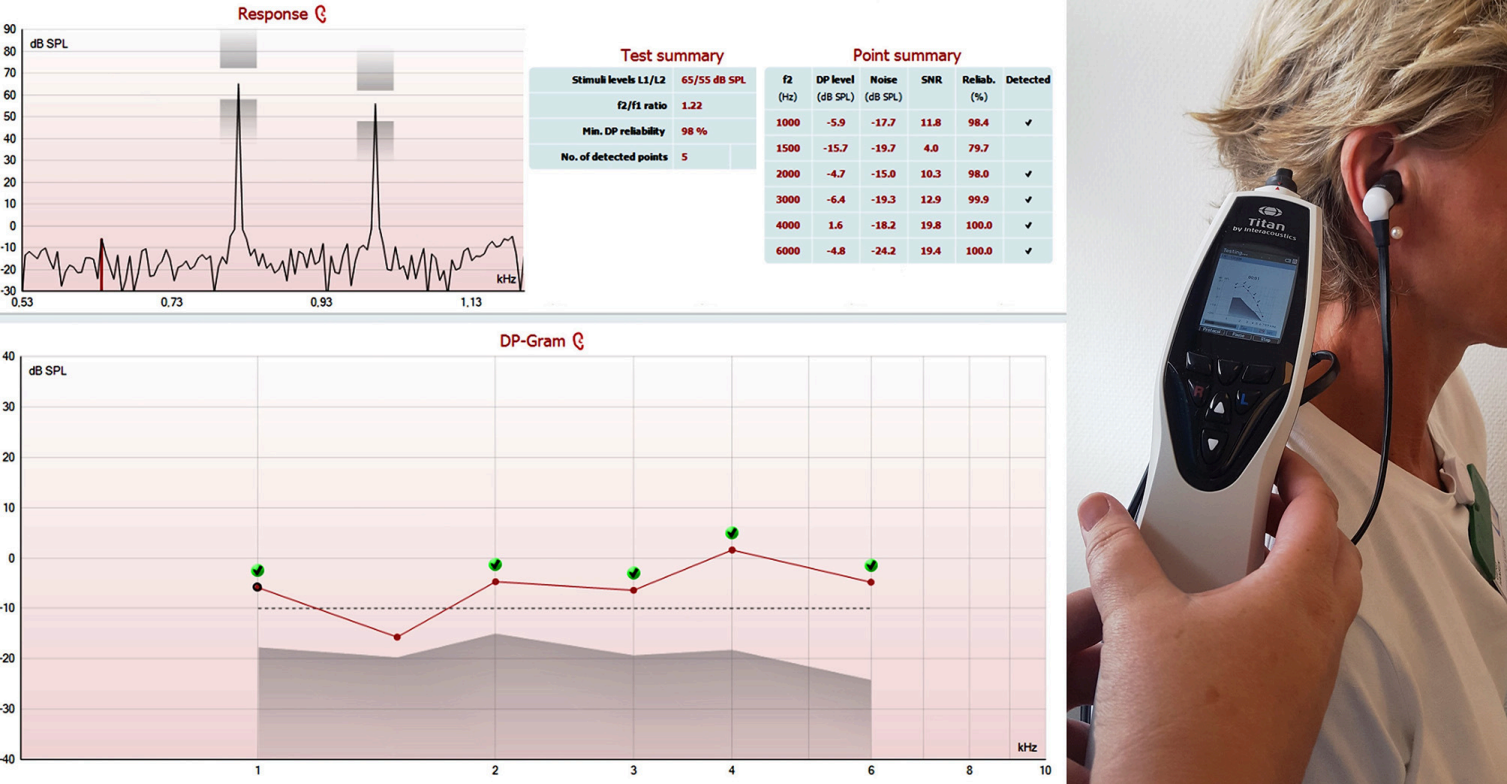
This figure shows a screenshot depicting a schematic overview of distortion product otoacoustic emissions (DPOAE) of the right ear of a healthy volunteer. DPOAE were present at 1, 2, 3, 4, and 6 kHz (but not 1.5 kHz). Otoacoustic emissions were assessed with the OAE Titan (®Interacoustics, Middelfart, Denmark), an automated handheld device. The procedure takes less than 2 min. dB SPL – decibel sound pressure level; kHz −1,000 Hertz.

A pre-neuronal phenomenon, otoacoustic emissions are unaffected by sedation; they can be assessed non-invasively at the bedside using an automated hand-held device; costs are low; and analysis does not require elaborate data post-processing (Figure 1) (1, 2). These are all features of a convenient candidate biomarker for prognostication following anoxic brain injury. However, the usefulness of otoacoustic emissions as prognostic markers after cardiac arrest is unknown.

The intention of this exploratory study was to compare otoacoustic emissions in patients from the extreme sides of the clinical spectrum, that is, cardiac arrest with poor neurological prognosis (fatal cerebral anoxic-ischemic injury) on one side and myocardial infarction without loss of consciousness on the other (no cerebral anoxic-ischemic injury).

We hypothesized that otoacoustic emissions would be absent in comatose cardiac arrest patients with irreversible anoxic-ischemic encephalopathy but relatively preserved in neurologically normal patients with myocardial infarctions and without prior loss of consciousness.

## Methods

We assessed distortion product otoacoustic emissions (DPOAE) and transient evoked otoacoustic emissions (TEOAE) in both ears of 10 consecutive unresponsive cardiac arrest survivors in whom a decision had been made to withdraw treatment based on standardized neurological assessment, including neuroimaging, electroencephalography, median nerve sensory evoked potentials, and serum biomarkers, ≥ 72 h after target temperature management and tapering of sedation. DPOAE and TEOAE, as well as auditory brainstem response audiometry, were assessed within 3 h prior to extubation and palliation. Ten age- and sex-matched patients with myocardial infarction without prior loss of consciousness served as controls.

Otoscopy and tympanometry was performed prior to testing of otoacoustic emissions in order to exclude obstruction of the ear canal and middle-ear effusion. Otoacoustic emissions were assessed using OAE Titan (®Interacoustics, Middelfart, Denmark) and TEOAE were recorded using AccuScreen (®Otometrics, Taastrup, Denmark), as described earlier (1). Briefly, a sensitive, low noise microphone is sealed in the external ear canal and an acoustic stimulus is delivered. The sound in the external ear canal that is elicited in response to the acoustic stimulus is recorded by the microphone (Figure 1).

Odds ratios were calculated and the level of statistical significance was set to *p* < 0.01. The Ethics Committee of the Capital Region of Denmark (De Videnskabsetiske Kommiteer—Region Hovedstaden, Hillerød, Denmark) approved the study and waived the need for written consent because risks were deemed negligible (reference j.nr. H-17038640).

## Results

Following cardiac arrest with severe anoxic-ischemic brain injury, TEOAE were present in 2 out of 20 measurements (i.e., 2 out of 10 cases; mean age 63.2 ± 12.6 years; 2 females). Following myocardial infarction without loss of consciousness, TEOAE were noticed in 9/20 measurements (5/10 controls; mean age 66.5 ± 8.3 years; 2 females).

DPOAE (which are frequency-specific) were present in 11/120 measurements after cardiac arrest (4/10 cases) and in 49/120 measurements following myocardial infarction (9/10 controls).

Compared to myocardial infarct patients with preserved consciousness, cardiac arrest patients had significantly less often preserved DPOAE [9.2 vs. 40.8%; OR 0.15 (CI 0.07–0.30); z statistic 5.24; *p* < 0.0001)].

Table 1 provides further details.

**Table 1A:** Contingency table showing the frequency of present vs. absent distortion product otoacoustic emissions (DPOAE) in patients following cardiac arrest (cases), respectively, myocardial infarction without loss of consciousness (controls).

	**DPOAE present**	**DPOAE absent**	**Total**	**Percent**
*Cases* Post-cardiac arrest patients (*n* = 10)	11	109	120	9.2%
*Controls* Myocardial infarct patients (*n* = 10)	49	71	120	40.8%

*DPOAE were significantly less frequent after cardiac arrest (9.2 vs. 40.8%; OR 0.15 (CI 0.07–0.30); p < 0.0001)*.

## Discussion

Outcome prognostication following cardiac arrest is essential, yet challenging (3). EEG is valuable but affected by levels of sedation and requires neurophysiological expertise (4, 5). Similarly, magnetic resonance imaging is promising but is associated with significant logistical challenges in the intensive care setting (6). Finally, new biomarkers such as serum tau appear to have good sensitivity and specificity but need further validation (7). A cheap point-of-care test that is easily interpretable, universally available and unaffected by sedation is clearly needed.

Otoacoustic emissions fulfill all criteria mentioned (1, 2) but previous studies have only assessed their role in perinatal anoxic-ischemic injury (8–10) and intracranial hypertension (11–13). Our study is the first to assess the potential of otoacoustic emissions for prognostication of adult cardiac arrest survivors. However, we conclude that otoacoustic emissions do not represent reliable outcome markers for neurological recovery after cardiac arrest because we identified 4 cases with (partially) preserved otoacoustic emissions in a sample of 10 unresponsive post-cardiac arrest patients whose neurological condition was so poor that active treatment was withdrawn (cases 1, 5-7; Table 1b).

**Table 1B:** Summary of individual results from cardiac arrest patients (cases) and patients with myocardial infarction but normal consciousness (controls).

	**Preserved responses after cardiac arrest (*n* = 10 cases)**	**Preserved responses after myocardial infarction (*n* = 10 controls)**
Auditory brainstem response audiometry 45 dB nHL	1r,l; 2l	3r,l; 5r,l; 6r,l; 7r,l; 8r,l; 10r,l
TEOAE	1r; 5l	2r,l; 3l,r; 6r,l; 8r; 10r,l
DPOAE 1 kHz	6l	2l; 5l,r; 7r; 8r; 10r,l
DPOAE 1.5 kHz	1r; 5l; 6l	2r,l; 3r,l; 5r,l; 6r,l; 8r,l; 9r; 10r,l
DPOAE 2 kHz	1r, 6l, 7r	2r; 3r,l; 4r; 5r; 6l,r; 8r,l; 9r; 10r,l
DPOAE 3 kHz	1r, l	3r,l; 6r; 7r,l; 8r,l; 9r;10r,l
DPOAE 4 kHz	1r; 6r	3r,l; 4r; 7r,l; 8r; 9r
DPOAE 6 kHz		

*Only preserved responses are listed. Auditory brainstem responses could not be acquired in 3 controls due to excessive facial hair growth (patient 4), signal loss for technical reasons (patient 2), and complaints of lightheadedness (patient 9), respectively. DPOAE, distortion product otoacoustic emissions; l, left ear; r, right ear; TEOAE, transient evoked otoacoustic emissions; numbers denote individual patients. dB nHL, decibel normalized hearing level*.

While isolated measurements do not appear to add crucial information at the single-subject level, our results indicate, however, that otoacoustic emissions (i.e., DPOAE) are still affected by global anoxia following cardiac arrest (*p* < 0.0001). Thus, their usefulness as part of a multimodal approach, including serial measurements, should be further investigated. Decaying outer hair cell function over time may serve as a proxy for evolving ischemic brain damage.

## Author contributions

DK: study concept, acquisition of data, analysis and interpretation, writing of the manuscript, critical revision for important intellectual content, and approval of final manuscript. AJ, TH, and MB: acquisition of data, critical revision for important intellectual content, and approval of final manuscript. JK: study concept, acquisition of data, analysis and interpretation, critical revision for important intellectual content, and approval of final manuscript.

### Conflict of interest statement

The authors declare that the research was conducted in the absence of any commercial or financial relationships that could be construed as a potential conflict of interest. The reviewer CD and the handling editor declared their shared affiliation.
